# Advances and Environmental Conditions of Spring Migration Phenology of American White Pelicans

**DOI:** 10.1038/srep40339

**Published:** 2017-01-16

**Authors:** D. Tommy King, Guiming Wang, Zhiqiang Yang, Justin W. Fischer

**Affiliations:** 1 U. S. Department of Agriculture, Wildlife Services, National Wildlife Research Center, P.O. Box 6099, Mississippi State, Mississippi 39762, USA; 2 Department of Wildlife, Fisheries and Aquaculture, Mail Stop 9690, Mississippi State University, Mississippi State, Mississippi 39762, USA; 3 Department of Forest Ecosystem and Society, Oregon State University, Corvallis, Oregon 97331, USA; 4 U. S. Department of Agriculture, Wildlife Services, National Wildlife Research Center, Fort Collins, Colorado 80521, USA

## Abstract

Spring migration phenology of birds has advanced under warming climate. Migration timing of short-distance migrants is believed to be responsive to environmental changes primarily under exogenous control. However, understanding the ecological causes of the advancement in avian spring migration phenology is still a challenge due to the lack of long-term precise location data. We used 11 years of Global Positioning System relocation data to determine four different migration dates of the annual migration cycle of the American white pelican (*Pelecanus erythrorhynchos*), a short-distance migrant. We also tested the hypothesis that increases in winter temperature and precipitation on the wintering grounds would advance pelican spring migration. Pelican spring departures and arrivals advanced steadily from 2002 to 2011. Spring departure timing exhibited high repeatability at the upper end of migration timing repeatability reported in literature. However, individual spring departure and arrival dates were not related to winter daily temperature, total winter precipitation, and detrended vegetation green-up dates indexed by the normalized difference vegetation index. Despite high repeatability, the observed between-year variation of spring departure dates was still sufficient for the advancement of spring departure timing.

Advancements in spring migration phenology due to climate change have been extensively documented in birds^1^,^2^,^3^,^4^. A growing body of evidence suggests that local temperatures directly or indirectly fine-tune the migration phenology of birds^4^. Short-distance migrants have arrived at the breeding grounds earlier with increasingly higher ambient temperatures at the non-breeding grounds^4^,^5^,^6^. Timely spring arrival at the breeding grounds may enhance nesting opportunities and reproductive success of migrants^1^. Migrants may use endogenous (e.g., physiological) mechanisms to control the timing of spring migration, particularly in long-distance migrants^7^. However, high responsiveness of migration timing to climate change may enable migratory birds, particularly short-distance migrants, to arrive at the breeding grounds with favourable climate conditions and resources needed for reproductive success^1^,^3^. Nevertheless, the relative importance of endogenous and exogenous (e.g., phenotypic plasticity) mechanisms for the advancement of spring migration phenology are still elusive^2^.

Advancements in the spring migration phenology of birds may result from the microevolution and (or) the phenotypic plasticity of spring migration timing^8^,^9^. The microevolution hypothesis predicts that the spring migration phenology of bird populations would advance if natural selection shifts the peak of allele frequency distributions towards genes for the early onset of spring migration^8^. Repeatability estimates the upper limit of the heritability of phenotypic traits as the proportion of inter-individual or intra-class variance of phenotypic traits in total phenotypic variance^10^,^11^. If between-individual differences reflect inter-individual genetic differences, high repeatability may suggest genetic control, providing insight into the microevolution of avian migration timing^3^,^12^. Estimation of avian migration timing repeatability has been hindered by lack of accurate, repeated observations of migration timings until recent advances in global positioning system (GPS) tracking technology.

Phenotypic plasticity of life history traits may allow birds to track novel environments rapidly^3^,^8^. Fine-grained phenotypic responses to environmental fluctuations at population and individual levels may suggest phenotypic plasticity because microevolution is not fine-tuned to relatively short-term environmental changes^13^. Furthermore, ontogenic or age-related variation in individual migration timing is another form of phenotypic elasticity. Saino *et al*.^14^ found that experienced barn swallows (*Hirundo rustica*) also arrived at the breeding grounds earlier than inexperienced birds. Ageing may enhance physiological conditions and migration experiences (i.e., via learning) prompting older individuals to arrive at the breeding grounds earlier than at younger ages.

Avian migrants may track spatiotemporal variation in the phenology and availability of food en route. The normalized difference vegetation index (NDVI) has been widely used to measure or monitor variation in the environmental conditions of wildlife habitat^15^. Temporal variation in the NDVI in the eastern Sahel during winter explained about 88% of the total variance in the annual juvenile survival of migratory, carnivorous white stork (*Ciconia ciconia*) breeding in Poland^16^. Studies have shown that ecological productivity phenology, indexed by the NDVI, affects the migration phenology of insectorous birds^14^,^17^,^18^. The NDVI has also been used to monitor photosynthetic activities of floating and emergent plants in northern temperate wetland and the health of coastal wetlands^19^,^20^. Abundance of salamanders (*Salamandra slamandra*) was positively related to spatial variation in the NDVI among 17 streams in Switzerland^21^. It is plausible to expand the role of NDVI to index the ecological conditions and phenology of ecosystems, beyond a mere surrogate of food availability. We hypothesized that advanced primary productivity phenology on the non-breeding grounds would lead to early spring departures of avian migrants including fish-eating birds, which we termed the ecological productivity phenology hypothesis^18^.

The American white pelican (*Pelecanus erythrorhynchos*; hereafter, pelican) is the largest native bird of North America with body mass ranging from 4.1 to 14 kg^22^. The pelican is mainly distributed in western and central North America. The North American Continental Divide separates the range of the pelican into relatively distinct eastern and western sub-ranges^22^,^23^,^24^. The pelicans in the eastern sub-range are short-distance migrants: breeding mainly in the Northern Great Plains and wintering in the lower Mississippi River Valley and along the Gulf of Mexico^25^,^26^. Pelicans primarily select wetlands and aquaculture facilities as wintering habitat and feed on fish and salamanders in the Northern Gulf of Mexico^25^,^27^. Increases in winter temperature and precipitation may enhance emergent herbaceous wetlands and prey abundance of the pelicans in spring. Fall migration is thought to be temperature driven (e.g., freezing of water bodies on the northern breeding ranges)^22^. Spring arrival at the breeding grounds at Chase Lake, North Dakota, USA has advanced over the past three decades; however, environmental drivers and evolutionary mechanisms underlying the advancement are still unknown^28^.

In this study, we used 11 years of Global Positioning System (GPS) relocation data of 36 pelicans to determine whether the advancement of pelican spring migration phenology resulted from phenotypic plasticity. First, we determined four different spring and autumn migration timings during the annual migration cycle of pelicans and estimated the repeatability of the different migration timings, taking advantage of mutiple years of repeated observations of the same individuals. Second, we tested the prediction that increases in winter temperatiure and precipitation of the wintering grounds would advance spring migration of the pelicans. Last, we tested the prediction of the ecological productivity phenology hypothesis that pelicans would fine-tune spring departure timing based on the phenological phases of primary productivity at the non-breeding grounds. Emergent herbaceous wetlands constitute about 11% of pelican winter home range and 24% of habitat used by pelicans in the Northern Gulf of Mexico (T. King, unpublished data). It is plausible to assume that the vegetation phenology is an environmental cue of spring departures of pelicans^1^,^21^,^29^. Understanding the repeatability and responses of the migration timing of large-sized birds has important implications for the conservation and management of large-sized birds under predicted global warming.

## Results

**Figure 1:**
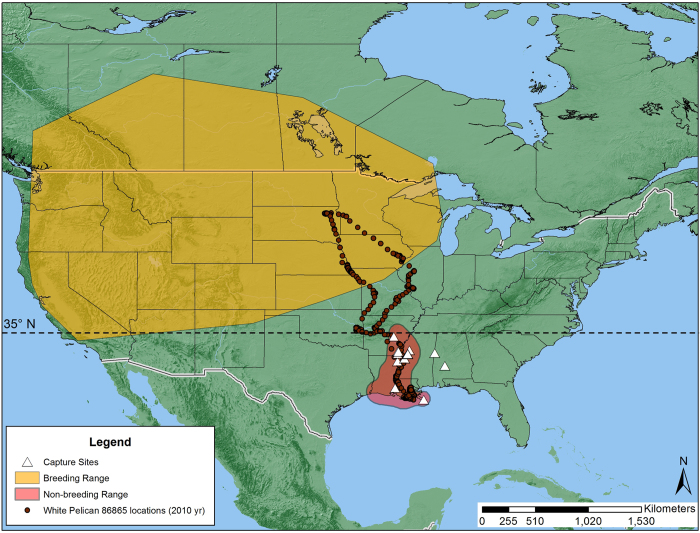
Breeding range (gold convex hull polygon), non-breeding range (red polygon), and spring capture locations (white triangles) of American white pelicans (*Pelecanus erythrorhynchos*) in the Southeastern United States, 2002–2012. The dots illustrate the locations during the 2010 annual migration cycle of pelican #86865. The map was generated using ArcGIS 10.2 software and its basemap (Environmental Systems Research Institute, Redlands, CA, USA; https://www.arcgis.com/home/webmap/viewer.html).

**Figure 2:**
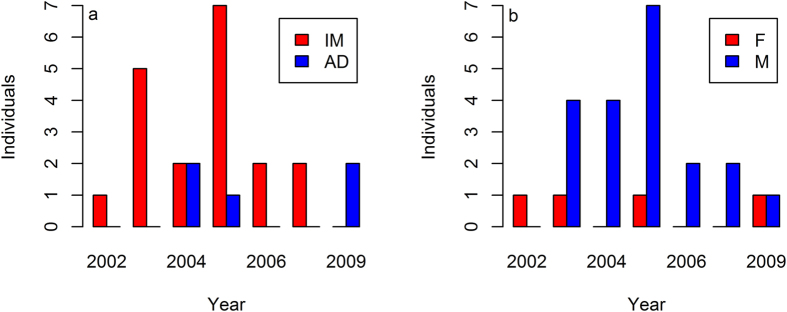
Annual (**a**) age and (**b**) sex composition of American white pelicans (*Pelecanus erythrorhynchos*) captured in Alabama, Louisiana, and Mississippi, USA from 2002 to 2007 and 2009. Symbol “IM” stands for immature, “AD” for adult, “F” for female, and “M” for male.

Thirty six birds, wintering along the Gulf of Mexico Coast and the Lower Mississippi River Valley in Louisiana and Mississippi from 2002 to 2011, were used in this study. The GPS locations indicated that 12 birds were year-round residents in Arkansas, Louisiana, and Mississippi. These 12 birds (*n* = 10 from 2002 to 2005; *n* = 2 from 2009–2011) were tracked for 9–32 months with a range of 2–24 relocations per day. Twenty four of the 36 birds migrated between the wintering grounds primarily in Louisiana and Mississippi and the breeding grounds in the Northern Great Plains (Fig. 1). Twelve of the 24 migrating birds were tracked for 10 to 12 consecutive months, 11 birds for 18 to 24 months, and one bird for 48 consecutive months. The majority of annual captures were immature males (Fig. 2a). Migrating pelicans comprised 20 males and four females (Fig. 2b).

Migrating pelicans tracked in this study spent about six months of winter in the Northern Gulf of Mexico from October through the following April, departed from the wintering grounds for the breeding grounds in spring, spent about three months of summer (June, July, and August) at the breeding grounds in the Northern Great Plains, and returned to the wintering grounds in October (Fig. 1, Table 1). The repeatability of migration timing varied between the four different dates of the annual migration cycle, with the repeatability of spring departures being high (Table 1). Differences in spring departures of the same individuals between successive years ranged from 3 to 35 days, averaging 17.27 days.

**Figure 3:**
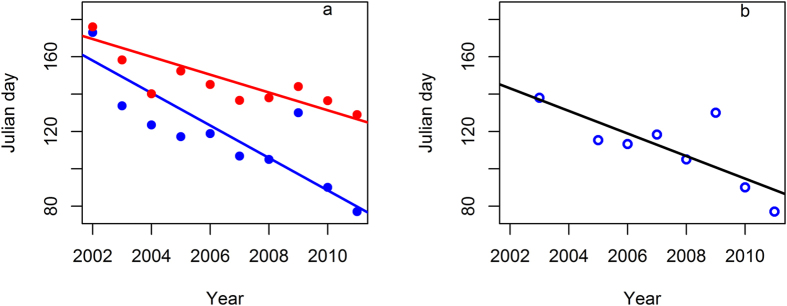
Advanced timings of (**a**) population mean spring departure (blue dots and line) from the non-breeding grounds and spring arrival at the breeding grounds (red dots and line) and (**b**) adult mean spring departure of American white pelicans (*Pelecanus erythrorhynchos*), wintering in Louisiana and Mississippi, USA from 2002 to 2012.

Pelican population spring departures advanced from 2002 to 2011 (slope [*β*] = −6.44 day yr^−1^, AICc_null_ = 93.51, AICc_year_ = 84.47, *n* = 10, Fig. 3a), and so did population spring arrivals (*β* = −3.10 day yr^−1^, AICc_null_ = 81.87, AICc_year_ = 78.19, *n* = 10, Fig. 3a). Average spring departures of adult pelicans also advanced over years (*β* = −5.45 day yr^−1^, AICc_null_ = 74.29, AICc_year_ = 71.81, *n* = 8, Fig. 3b). Linear models for the data of annual average immature and adult spring departure dates did not support an interaction between year and age at the population level (AICc_null_ = 128.97, AICc_age_ = 130.05, AICc_year_ = 118.43, AICc_age+year_ = 122.43, AICc_age*year_ = 125.44, *n* = 14). Vegetation green-up dates advanced pronouncedly at the non-breeding grounds from 2001 to 2011 (β = −1.61day yr^−1^, AICc_null_ = 78.79, AICc_green-up_ = 73.61, *n* = 11). Nevertheless, neither winter daily temperature (AICc_null_ = 38.98, AICc_temp_ = 41.87) nor total winter precipitation (AICc_null_ = 79.37, AICc_prec_ = 83.09) had linear temporal trends from 2002 to 2012.

**Figure 4:**
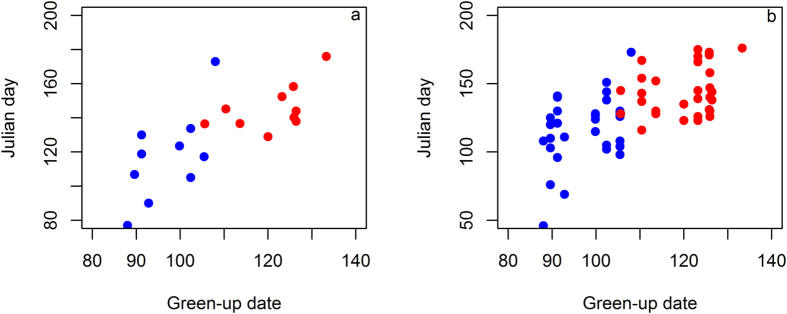
Paralleled increasing trends of (**a**) population mean spring migration dates and (**b**) individual spring migration dates of American white pelicans (*Pelecanus erythrorhynchos*) with vegetation green-up dates from 2002 to 2011. Blue dots are the data on the non-breeding grounds, and red dots are for the breeding grounds.

Neither spring departures nor spring arrivals were related to winter daily temperatures or total winter precipitation on the breeding ground in the longitudinal analysis with LMMs including a term of year (Tables S2–S3). Although the LMM including year, age, and winter mean daily temperature was the most parsimonious model for both spring departures and arrivals, estimation precision, measured by standard error, was too low to be conclusive for the temperature effects on both the migration dates (Supplemental Tables S2–S3). Increasing trends in individual spring migration dates paralleled vegetation green-up dates (Fig. 4). However, detrended spring departure and arrival dates were not related to detrended green-up dates in the respective longitudinal analysis (Supplemental Tables S4–S5).

## Discussion

The migration phenology of short-distance migrants is controlled primarily by exogenous factors^7^,^30^. Highly repeatable spring departure timing may signal some endogenous mechanism(s) for the individual-level circannual consistency of spring migration initiation of American white pelicans, one of the largest flying birds. On the contrary, low repeatability of spring arrivals and autumn departures suggest environmental causes for the variation in the migration phenology of the pelican^3^. However, we did not find evidence supporting our hypotheses concerning the effects of climatic changes and vegetation green-ups at pelican wintering grounds on spring departure and arrival timings. On the other hand, significant advances in the spring departures of both populations and adults suggest that age and migration experience may not be the only cause of the observed advancement of pelican spring migration.

Avian migration timing has the lowest repeatability (*r*) in a set of 12 behavioral traits analyzed by Bell *et al*.^31^. Average repeatability of animal behaviors analyzed in Bell *et al*.^31^ is 0.37 *(n* = 759). Nevertheless, Thorup *et al*.^12^ found that migratory passerines of northern Europe had significant repeatability (*r* = 0.2–0.5) of spring or autumn migration timing. Bar-tailed godwits (*Limosa limosa baueri*) exhibit consistent spring departure schedules (*r* = 0.83), leaving the wintering habitat in New Zealand in the same week of each year^32^. The spring departure date of pelicans from the non-breeding grounds had high repeatability (*r* = 0.76). Fecundity selection may result in the high repeatability of the spring departure of pelicans^33^. Autumn migrants are subjected to viability selection for survival through autumn and winter^34^. Autumn departure timing from the breeding grounds may depend not only on weather conditions, but also on the reproductive experience of migrant birds; thus, the autumn departure had the lowest repeatability during the entire annual migration cycle of the pelican. The date of autumn arrival in the non-breeding grounds had moderate repeatability compared to average repeatability of animal behaviors and those of migratory passerines. Departures before freezing of water bodies from the breeding range in the Northern Great Plains, known for variable, prolonged cold winters, may reduce the en route mortality of pelicans^28^,^35^. The significant repeatability of spring departure suggests that endogenous mechanisms may in part determine pelican migration timing.

Song birds and insectivorous raptors in the habitat of higher food availability and more rainfall depart earlier than those in the habitat of lower food availability and less rainfall^36^,^37^. The NDVI has been used to represent the ecological and habitat conditions of wintering and stopover habitats to predict the migration dates of terrestrial insectivorous birds^14^,^18^,^38^. Barn swallows (*Hirundo rustica*) advance spring arrival timing in Spain in response to changes in NDVI in the wintering habitat in North Africa^38^. Greater NDVI values at the spring departure time and during the entire winter stay in western Africa were shown to advance spring departure of the white stork, a carnivorous bird^39^. However, our results corroborated the conclusion of Gordo *et al*.^39^ that the importance of the NDVI during the entire wintering stay was confounded by the strong effect of year in their study. Schlaich *et al*.^37^ found that Montagu’s harriers (*Circus pygargus*) increased flying and foraging time in the habitat of low NDVI and grasshopper (prey) abundance and subsequently delayed spring departures from the wintering grounds in the Sahel. Despite the statistically spurious correlation revealed in this study, understanding respective ecological mechanisms underlying the temporal trends of vegetation green-up and spring migration timings may provide insights into the observed biological trends of migration phenology and ecological productivity phenology.

Individual responsiveness to environmental changes (i.e., phenotypical plasticity) may lead to the advancement of avian spring migration phenology. Microevolutionary or genetic responses to environmental changes are too coarse-grained to explain individual- or population-level responses to the stochastic fluctuation of environmental conditions^13^. Nevertheless, no evidence showed that the pronounced advancement in spring migration phenology was related to climatic changes in this study. Pelicans also have advanced spring arrival at Chase Lake, North Dakota, USA, and the advancement is not temperature related^28^. Buskirk *et al*.^40^ found that phenotypic plasticity only explains <25% of the temporal trend of the spring arrival timings of 27 bird species in Pennsylvania, USA during a 46-year period. They suggest that microevolution may be responsible for spring arrival advances observed in the birds studied^40^. Gill *et al*.^41^ recently proposed that spring migration dates of bird populations would advance if recruits of recent years are early birds starting spring migration earlier than those of previous years, without invoking phenotypic plasticity. Although populations of Icelandic black-tailed godwits (*Limosa limosa islandica*) advanced their spring arrival dates, the spring arrival dates of individuals have remained highly repeatable over years^41^. Phenotypic variation of spring departures (i.e., mean difference in the spring departure of the same tracked bird between two successive years) was greater than the predicted annual advancement of spring departures (17 days year^−1^ vs. about 7 days year^−1^). Therefore, despite high repeatability, the phenotypic variability of the spring departure exhibited in our observations is sufficient to allow pelicans to adjust their short-distance spring migration timing.

In summary, the migration timing of American white pelicans advanced substantially from 2002 to 2011. However, environmental causes of the spring migration advancement were still ellusive. Highly repeatable spring departure dates and moderately repeatable autumn arrival dates may suggest that endogenous mechanisms in part control the migration timing of pelicans if significant inter-individual differences in migration timing reflect genetic differences. However, long-term and large data are needed to evaluate the genetic heritability of the migrating time of long-lived species.

## Methods

### Capture and GPS tracking of pelicans

We captured pelicans at loafing sites near aquaculture-intensive areas in Alabama, Arkansas, Louisiana, and Mississippi, USA (Fig. 1) using rocket nets and modified foot-hold traps during March and April from 2002 to 2009 42. The map of Fig. 1 was generated using ArcGIS 10.2 software and its basemap (Environmental Systems Research Institute, Redlands, California, USA; https://www.arcgis.com/home/webmap/viewer.html). We estimated age (>3 yr old = adult; <3 yr old = immature) by plumage and eye and skin color characteristics (T. King, unpublished data) and sex by culmen length for each captured pelican^43^. Captured pelicans were fitted with 70-g solar-powered GPS satellite transmitters (<3% of the bird’s body mass; PTT-100, Microwave Telemetry, Columbia, Maryland, USA) using a backpack harness^44^. The location error of the GPS was about + 18 m. Transmitters were programmed to record one location per hour for the duration of the study. Handling time of each captured bird was about 10 minutes. We released all birds at the capture site after handling.

### Ethic statement

This research was conducted in accordance with the USDA guidelines of animal care and use. All experimental protocols of animal capture and handling were approved by the United States Department of Agriculture (USDA), National Wildlife Research Center, Institutional Animal Care and Use Committee (IACUC Protocol QA-1018).

### Estimation of non-breeding range, summer range, and breeding range

Pelicans have expanded their wintering range northward from the Gulf of Mexico coast into areas with commercial aquaculture facilities, primarily due to the development of channel catfish (*Ictalurus punctatus*) aquaculture in the region^25^. To determine the arrival dates in and departure dates from the non-breeding range, the species’ breeding range, and individual summer home range, we first estimated the boundaries of the three ranges. We combined all relocations of the 12 year-round residents in the Northern Gulf of Mexico, and estimated their “super” home range using the 95% kernel smoothing methods in the R package *adehabitatHR* (http://cran.r-project.org/web/packages/adehabitatHR)^45^. We used the super home range of the resident birds as the non-breeding range^46^.

To determine the relocations within summer range, we plotted the net squared displacement distance of a migrating pelican over time (month, day, and hour) for all hourly locations during a calendar year. The net squared displacement distances are the squared distances between the initial location in the non-breeding range and subsequent locations^47^. Simulation and empirical studies have shown that net squared displacement of migrants varies over time following a double logistic curve: increasing after the onset of spring migration; leveling off after arrival in summer range; and decreasing after the onset of autumn migration (Supplementary Fig. S1)^48^. Net squared displacement has been successfully used to estimate the migration timing and summer range of migratory ungulates tracked by GPS transmitters^49^,^50^. We used the R function *identify* to determine the start and end locations of the net squared displacement plateau. Then we used the *adehabitatHR* to estimate the 95% kernel summer home range of migrating pelicans, with all available hourly GPS relocations between the start and end locations. We used the 95% summer home range as the summer habitat of each migrating pelican. A “super” summer home range was also estimated using all locations within all individual summer habitats. We used 80% of the reference bandwidth as the smoothing factor in summer and winter home range estimation^51^.

We combined the geographic locations of known pelican colonies from King and Anderson^24^ and the shapefile of the summer relative abundance distribution map of pelicans from the North America Breeding Bird Survey (BBS) data archive (http://www.mbr-pwrc.usgs.gov/bbs/shape_ra10.html) to estimate the southern boundary of the breeding range. The breeding range was delineated by the smallest convex polygon or convex hull, covering all known nesting colonies and the BBS sites within the eastern and southern boundaries of the summer range, using the R function *chull* (Fig. 1).

### Determination of migration dates

We determined four dates during the annual cycle of pelican migration using the GPS location time series of migrating birds. The four migration dates included spring departure from the non-breeding range in the northern Gulf of Mexico area (spring departure), spring arrival in the summer range (spring arrival), autumn departure from the summer range (autumn departure), and autumn arrival in the non-breeding range (autumn arrival; Fig. 1). We determined the four dates by plotting the movement paths of migrating pelicans dynamically on a US map overlaid with summer and winter habitat or range boundary shape files (see Supplementary Map S1 and Supplementary material for the definitions of the four dates and the description of date determination methods).

### Normalized difference vegetation index and estimation of vegetation phenology

We used 250-m Moderate Resolution Imaging Spectroradiometer (MODIS) NDVI data to estimate vegetation phenology within the winter and summer super home range boundaries at the non-breeding and breeding grounds (https://lpdaac.usgs.gov/products/modis_products_table). We used program TIMESAT to estimate annual dates of vegetation green-up using the NDVI time series within the southern part (i.e., south of 31.5° N latitude) of the winter super home range (Fig. 1). The green-up date was estimated as the date when 50% of the difference between annual maximum and minimum NDVI (i.e., annual NDVI amplitude) was reached in spring^52^. The vegetation green-up in the southern wintering area was earlier than spring departure from 2001 through 2012. Vegetation green-up dates on the breeding grounds were also estimated using the NDVI time series from 2001 to 2012 within the summer super home range using TIMESAT.

### Climate variables

We used all weather stations located within the super winter home range (Fig. 1) to compile winter mean daily temperature (C°) and total winter precipitation (cm) from November 1 through January 1 of the following years from 2002 to 2012. The winter period was determined based on the regional climate and the fact that pelicans started spring migration in early February. The weather data were downloaded from National Oceanic and Atmospheric Administration (NOAA) National Centers for Environmental Information (http://www.ncdc.noaa.gov/cdo-web/datatools).

### Statistical analysis

We used the R package *rptR* to calculate the repeatability of spring departure, spring arrival, autumn departure, and autumn return with birds having > 1 year of GPS tracking data (http://rptr.r-forge.r-project.org/). We used the permutation test for the significance of repeatability (*n* = 5,000).

We conducted cross sectional analysis of spring departure and arrival using the population and adult average migration dates. We averaged the dates of spring departures and spring arrivals over all tracked birds by year for cross sectional analysis. We also averaged the dates of spring departures over adults (>3 years old). We used linear models (LMs) for cross sectional analysis after testing for the necessity of temporal autocorrelative error (see model selection for the order-1 temporal autocorrelation in support information; Table S1). If adult mean spring departures advanced over years, the effects of aging and migration experiences cannot explain the population-level advancement alone.

We used linear mixed models (LMMs) for the longitudinal analysis of the effects of winter climate and green-up timing on an individual’s spring departure and arrival, respectively, with animal identification (ID) as a random effect. Year was included as a covariate in all LMMs for climatic effects because of the pronounced yearly trend in the migration dates. The full model including all covariates was of the form: date~year + age + temp + prec + temp*prec + (1|ID), where temp is winter mean daily temperature, prec is total winter precipitation, and ID is individual identification as a random effect. To account for the effects of age, we determined the age (i.e., immature or mature) of migrating individuals for each migration year. We built LMMs to include all possible combinations of climate variable or green-up date and age (i.e., year only, year + age, year + climate variable, and year + age + interaction between climate variables). We used information-theoretic approaches to model selection. The best model has the lowest corrected Akaike information criterion (AICc) value. Competing models have the ΔAICc values < 2.0^53^.

To test for potential spurious correlation between spring migration timing and green-up timing, we detrended spring migration dates (i.e., spring departure and arrival dates) and green-up dates by season using linear regression with year as a covariate. We applied LMMs to the detrended migration dates as a response variable and detrended green-up dates as a covariate for both longitudinal analyses. Similarly, LMM with an intercept only was used as a null model and compared to the LMMs of all possible combinations of detrended green-up date, age, and interaction between age and detrended green-up. Thus, the full model including all covariates had the form of: ddate ~ dgreenup + age + dgreenup*age + (1|ID), where ddate is detrended migration date, and dgreenup is detrended green-up date. All LMMs were built with the R package *lme4*^54^,^55^. All statistical analyses were conducted in the R 3.0.2 environment (R Development Core Team, 2012).

## Additional Information

**How to cite this article**: King, D. T. *et al*. Advances and Environmental Conditions of Spring Migration Phenology of American White Pelicans. *Sci. Rep.* **7**, 40339; doi: 10.1038/srep40339 (2017).

**Publisher's note:** Springer Nature remains neutral with regard to jurisdictional claims in published maps and institutional affiliations.

## Supplementary Material

Supplementary Information

## Figures and Tables

**Table 1:** Migration dates and repeatability of the annual migration cycle of American white pelicans (*Pelecanus erythrorhynchos*) captured in Alabama, Louisiana, and Mississippi, USA from 2002 to 2009.

Migration date	Median date	Repeatability
Spring departure from non-breeding range	1 May	0.763 (0.001)
Spring arrival in summer habitat	24 May	0.318 (0.168)
Autumn departure from summer habitat	29 August	0.020 (0.478)
Autumn arrival in non-breeding range	4 October	0.367 (0.096)

Numbers in the parentheses after the repeatability are the p values.
