# Supplementary material for: Advances and Environmental Conditions of Spring Migration Phenology of American White Pelicans
Source: Sci Rep. 2017 Jan 16;7:40339. doi: 10.1038/srep40339 (PMC5238423; doi:10.1038/srep40339)
Supplement: Supplementary Information [file srep40339-s1.doc]

**Advances and Environmental Conditions of Spring Migration Phenology of American White Pelicans**

**D. Tommy King^1^,*, Guiming Wang^2^, Zhiqiang Yang^3^, and Justin W. Fischer^4^**

^1^U. S. Department of Agriculture, Wildlife Services, National Wildlife Research Center, P.O. Box 6099, Mississippi State, Mississippi 39762, USA; E-mail: Tommy.King@aphis.usda.gov

^2^Department of Wildlife, Fisheries and Aquaculture, Mail Stop 9690, Mississippi State University, Mississippi State, Mississippi 39762, USA; Email: guiming.wang@msstate.edu

^3^Department of Forest Ecosystem and Society, Oregon State University, Corvallis, Oregon 97331, USA; E-mail: zhiqiang.yang@oregonstate.edu

^4^U. S. Department of Agriculture, Wildlife Services, National Wildlife Research Center, Fort Collins, Colorado 80521, USA; E-mail: Justin.W.Fischer@aphis.usda.gov

**Supporting information:**

**Definitions of migration dates**

Spring departure from the non-breeding range is the first day when an American white pelican (*Pelecanus erythrorhynchos*; hereafter, pelican) crosses the non-breeding range boundary northward, continuing northward movement. Spring arrival in the summer habitat is the first day when a pelican arrives in its 95% kernel summer home range without immediately leaving the home range. The 95% home ranges account for some estimation uncertainty of the summer habitat area of migrating pelicans. Autumn departure from the breeding habitat is the first day when a pelican crosses the boundary of its 95% summer home range southward, continuing to move south. Autumn arrival in the non-breeding range is the first day when a migrating pelican crosses the northern boundary of the non-breeding range, continuing to move south.

Dates of migrants crossing the 35^o^ N latitude line northward during spring and departure dates from the non-breeding grounds were highly correlated (R^2^ = 0.98, d.f. = 30, P ≤ 0.01), and so were autumn arrival dates in the non-breeding range and dates crossing the 35^o^ N latitude line southward during autumn (R^2^ = 0.96, d.f. = 20, P ≤ 0.01). Therefore, we used the date crossing the 35^o^ N latitude line northward during spring as spring departure date and the date crossing the 35^o^ N latitude line southward during autumn as autumn arrival date in subsequent analyses.

**Methods for determining migration dates**

We used the function *as.ltraj* in the R *adehabitatLT* package to compute net squared displacement distances and the function *trajdyn* to display the movement trajectories dynamically (http://cran.r-project.org/web/packages/adehabitatLT). The non-breeding range polygon, the 35^o^ N latitude line, and migrating pelican summer home range polygons were overlaid on a map of the United States (US) to determine the migration dates to the nearest hour of a day. We then rounded down the migration times to Julian days for subsequent analysis.

Some migrating pelicans had missing GPS relocations for 1 to 5 days (< 2% of the tracking period) due to low battery voltages resulting from cloudy weather conditions. We fit continuous-time correlated random walk (CTCRW) models to the GPS relocations of each migrating pelican and predicted daily relocations in 24-hour intervals [^1^](#_ENREF_1). In a preliminary analysis, migration dates from hourly data and daily locations predicted by CTCRW models were consistent, with the R^2^ of linear regression of the former on the latter being 99%. If missing data occurred while a bird was crossing a boundary, we used the CTCRW to interpolate the daily missing relocation (i.e., one interpolated location per day) in the vicinity of the boundary lines to determine the migration dates.

**Model selection for the order-1 temporal autocorrelation**

To determine the necessity of autocorrelative error, we adopted information-theoretic model selection using corrected Aikaike information criterion (AICc) to compare generalized least square models (GLSs) with and without the order 1 temporal autocorrelation. If the AICc of GLSs of the year effect without temporal autocorrelation was less than that of GLSs with temporal autocorrelation by 2.0 or more, we used linear models (LMs) to regress population mean spring departure dates, adult mean spring departure dates, population mean spring arrivals, annual green-up dates, winter daily mean temperatures, and total winter precipitation against year, respectively, to test if spring migration dates, annual green-up dates, and winter climate had long-term temporal trends over years.

Figure S1. Temporal variation in the net squared displacement distance of migrating animals. The curve is depicted by a double logistic equation.


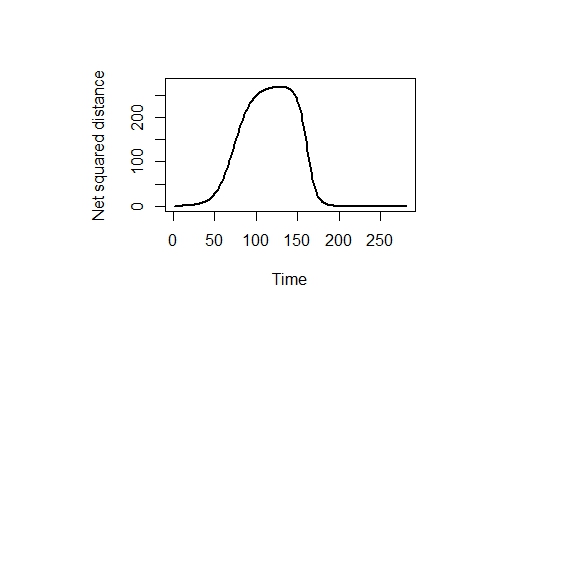


Figure S2. Scatter plots of (a) detrended individual spring departure dates and (b) detrended individual spring arrival dates of American white pelicans (*Pelecanus erythrorhynchos*) with detrended green-up dates from 2002 to 2011.


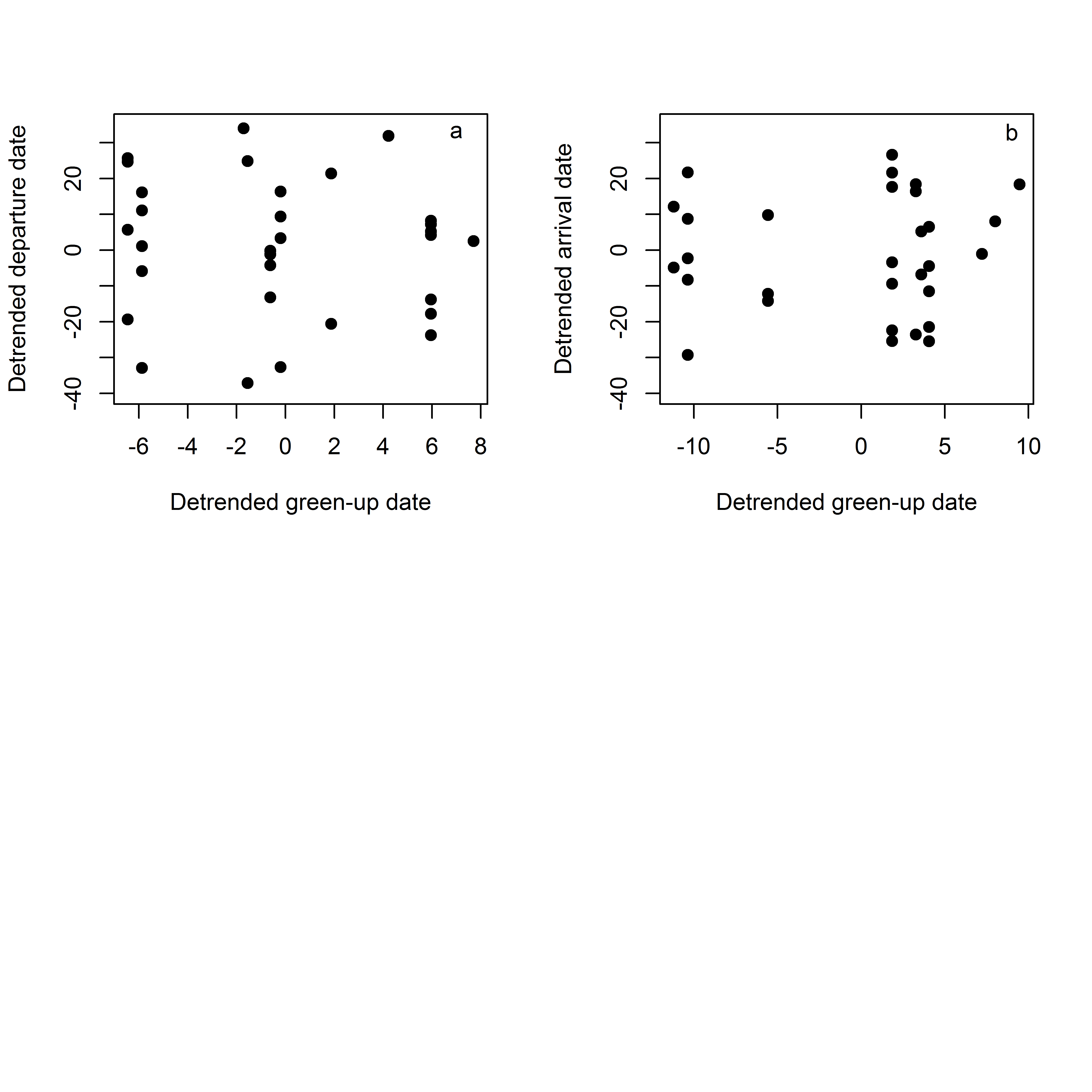


Map S1. Illustrating the 2010 annual migration cycle range boundaries of American white pelicans (*Pelecanus erythrorhynchos*) #86865. See the definition of migration dates in this appendix for a detailed explanation of spring departure (D1), spring arrival (D2), autumn departure (D3), and autumn arrival (D4). The map was generated using ArcGIS 10.2 software and its basemap (Environmental Systems Research Institute, Redlands, CA, USA; https://www.arcgis.com/home/webmap/viewer.html).


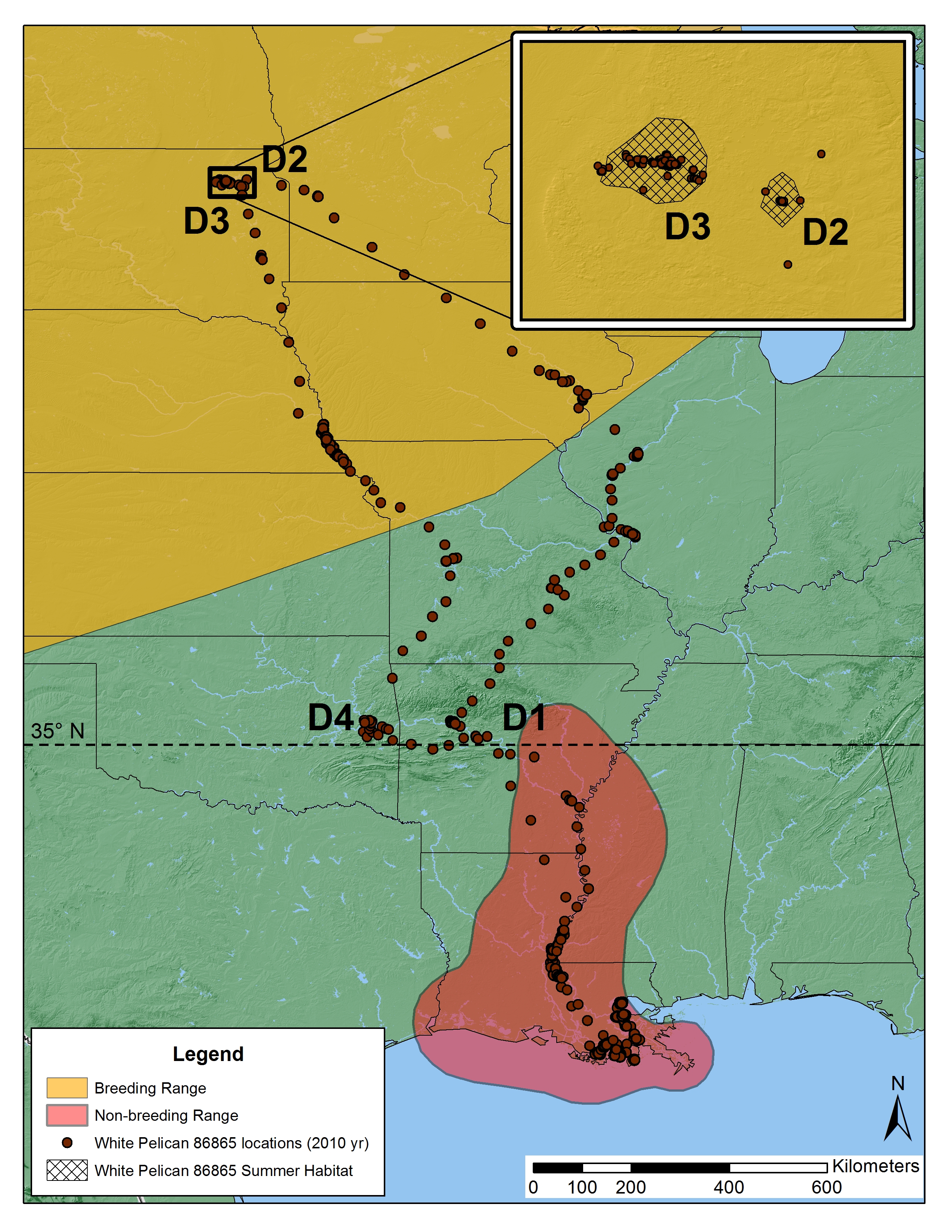


Table S1. Comparisons of generalized least squared models between the order-1 autocorrelative error (AR-1) and independent and identically distributed error (iid) for using Akaike information criterion (values in the cell) for the temporal (or year) trends of population mean spring departure date, adult mean spring departure, population mean spring arrival date, winter daily mean temperature at the wintering ground, total winter precipitation at the wintering ground, and vegetation green-up date at the wintering ground of American white pelicans.

| Error term | Population spring departure | Adult spring departure | Spring arrival | Daily temperature | Total precipitation | Green-up date |
| --- | --- | --- | --- | --- | --- | --- |
| iid | 83.35 | 66.64 | 73.09 | 44.88 | 78.6 | 70.84 |
| AR-1 | 88.64 | 75.87 | 77.89 | 50.88 | 82.86 | 75.53 |

Table S2. Linear mixed models for the effects of pelican age (adult and immature), winter mean daily temperature (temp), and total winter precipitation (prec) on individual pelican spring departure dates with individual identification as a random effect. Initial AICc stands for corrected Akaike information criterion. ΔAICc is difference in AICc between a model AICc and lowest AICc. Values in parentheses are standard error. Blank cells mean covariates were not included in a model.

| Model | AICc | ΔAICc | Slope of year | Slope of immature | Slope of temp | Slope of prec | Slope of temp*prec |
| --- | --- | --- | --- | --- | --- | --- | --- |
| year | 294.93 | 4.98 | -6.05 (1.70) |  |  |  |  |
| year+age | 291.80 | 1.85 | -6.05 (1.96) | 0.01 (7.67) |  |  |  |
| year+temp | 292.90 | 2.95 | -5.64 (1.8) |  | 2.47 (3.32) |  |  |
| year+prec | 297.48 | 7.54 | -6.05 (1.70) |  |  | -0.07 (0.45) |  |
| year+temp+prec | 295.67 | 5.72 | -5.67 (1.82) |  |  | -0.02 (0.45) | 2.43 (3.41) |
| year+temp*prec | 297.79 | 7.84 | -5.82 (1.78) |  | 17.96 (15.34) | 3.41 (3.35) | -0.43 (0.41) |
| year+age+temp | 289.95 | 0.00 | -5.57 (2.08) | 0.59 (7.51) | 2.49 (3.39) |  |  |
| year+age+prec | 294.52 | 4.57 | -6.08 (1.97) | -0.24 (7.82) |  | -0.08 (0.46) |  |
| year+age+temp+prec | 292.90 | 2.95 | -5.61 (2.10) | 0.46 (7.92) | 2.44 (3.49) | -0.02 (0.46) |  |
| year+age+temp*prec | 295.26 | 5.32 | -5.80 (2.07) | 0.27 (8.00) | 18.09 (15.68) | 3.43 (3.43) | -0.44 (0.42) |

Note: we fit two models of age-temp interaction and age-prec interaction, respectively. Although the AICc value is the lowest for the age-temp interaction model (i.e., AIC_cage*temp_ = 285.67, AICc_age*prec_ = 291.83), the estimate precision (e.g., standard error [SE]) of the interactive term coefficient was substantially large: β_adult.temp_ = -2.74, SE= 5.56; β_immature.temp_ = 10.15, SE= 8.64.

Table S3. Linear mixed models for the effects of pelican age (adult and immature), winter mean daily temperature (temp), and total winter precipitation (prec) on individual pelican spring arrival dates with individual identification as a random effect. Initial AICc stands for corrected Akaike information criterion. ΔAICc is difference in AICc between a model AICc and lowest AICc. Values in parentheses are standard error. Blank cells mean covariates were not included in a model.

| Model | AICc | ΔAICc | Slope of year | Slope of immature | Slope of temp | Slope of prec | Slope of temp*prec |
| --- | --- | --- | --- | --- | --- | --- | --- |
| year | 272.25 | 6.56 | -3.21 (1.45) |  |  |  |  |
| year+age | 268.03 | 2.33 | -2.02 (1.54) | 9.37 (6.70) |  |  |  |
| year+temp | 270.16 | 4.46 | -2.74 (1.62) |  | 2.80 (3.45) |  |  |
| year+prec | 274.64 | 8.95 | -3.34 (1.50) |  |  | 0.21 (0.45) |  |
| year+temp+prec | 272.59 | 6.89 | -2.83 (1.66) |  | 3.18 (3.52) | 0.30 (0.44) |  |
| year+temp*prec | 275.93 | 10.23 | -2.84 (1.67) |  | 4.10 (15.01) | 0.51 (3.24) | -0.43 (0.41) |
| year+age+temp | 265.69 | 0.00 | -1.15 (1.79) | 11.12 (7.32) | 3.60 (3.36) |  |  |
| year+age+prec | 270.67 | 4.97 | -2.05 (1.61) | 9.45 (7.13) |  | 0.08 (0.49) |  |
| year+age+temp+prec | 268.47 | 2.78 | -1.26 (1.84) | 11.04 (7.44) | 3.83 (3.46) | 0.19 (0.48) |  |
| year+age+temp*prec | 271.75 | 6.06 | -1.22 (1.84) | 11.95 (7.55) | 10.53 (16.34) | 1.70 (3.60) | -0.19 (0.44) |

Note: we fit two models of age-temp interaction and age-prec interaction, respectively. Although the AICc value is the lowest for the age-temp interaction model (i.e., AIC_cage*temp_ = 262.16, AICc_age*prec_ = 271.58), the estimate precision (e.g., standard error [SE]) of the interactive term coefficient was substantially large: β_adult.temp_ = -0.85, SE= 5.98; β_immature.temp_ = 7.33, SE= 8.11.

Table S4. Linear mixed model selection of the effects of detrended green-up date and bird age on detrended spring departure dates of American white pelicans (*Pelecanus erythrorhynchos*) using corrected Akaiki information criterion (AICc). Symbol ΔAICc denotes difference in AICc between a model AICc and lowest AICc. Values in the parentheses for regression slopes are standard errors.

| Model | AIC | ΔAIC | Slope of green-up date | Slope of immature | Slope of immature*green-up |
| --- | --- | --- | --- | --- | --- |
| Null | 295.27 | 3.05 | NA |  |  |
| green-up date | 296.89 | 4.66 | -0.27 (0.55) |  |  |
| age | 292.23 | 0.00 |  | -0.78 (6.56) |  |
| green-up date + age | 294.00 | 1.78 | -0.27 (0.60) | -0.65 (6.75) |  |
| green-up date*age | 292.43 | 0.21 | -0.78 (0.67) | -1.51 (6.68) | 2.07 (1.44) |

Table S5. Linear mixed model selection of the effects of detrended green-up date and bird age on detrended spring arrival dates of American white pelicans (*Pelecanus erythrorhynchos*) using corrected Akaiki information criterion (AICc). Symbol ΔAICc denotes difference in AICc between a model AICc and lowest AICc. Values in the parentheses for regression slopes are standard errors.

| Model | AIC | ΔAIC | Slope of green-up date | Slope of immature | Slope of immature*green-up |
| --- | --- | --- | --- | --- | --- |
| Null | 272.18 | 3.81 | NA |  |  |
| green-up date | 274.52 | 6.16 | 0.09 (0.45) |  |  |
| age | 268.36 | 0.00 |  | 6.46 (5.91) |  |
| green-up date + age | 270.89 | 2.53 | -0.01 (0.47) | 6.52 (6.12) |  |
| green-up date*age | 271.56 | 3.19 | 0.27 (0.58) | 5.80 (6.22) | -0.79 (0.98) |

**References**

1 Johnson, D. S., London, J. M., Lea, M. A. & Durban, J. W. Continuous-time correlated random walk model for animal telemetry data*. Ecolo*g**y** 89, 1208-1215 (2008).
